# The estimated incidence of lactational breast abscess and description of its management by percutaneous aspiration at the Douala General Hospital, Cameroon

**DOI:** 10.1186/s13006-020-00271-2

**Published:** 2020-04-10

**Authors:** Thomas Obinchemti Egbe, Theophile Nana Njamen, Henri Essome, Nicholas Tendongfor

**Affiliations:** 1 Faculty of Health Sciences, University of Buea, Box 63, Buea, Cameroon; 2 Department of Obstetrics and Gynecology, Douala General Hospital, P.O. Box 4856, Douala, Cameroon; 3 Faculty of Medicine and Pharmaceutical Sciences, University of Douala, Douala, Cameroon; 4 Department of Obstetrics and Gynecology, Laquintinie Hospital Douala, Douala, Cameroon

**Keywords:** Abscess, Breast, Breastfeeding, Drainage, Lactation, And mastitis

## Abstract

**Background:**

Lactational breast abscesses are uncommon in the puerperium but when they do develop, delays in specialist referral may occur especially in resource low settings. There is a dearth of studies regarding lactational breast abscesses in Cameroon. We aimed to estimate the incidence of lactational breast abscess and describe its management by percutaneous aspiration at the Douala General Hospital, Cameroon.

**Methods:**

We conducted an observational prospective study of 25 breastfeeding women at the Douala General Hospital from January 1, 2015, to October 31, 2015. Participants were consenting breastfeeding women who completed a baseline questionnaire after diagnosis of lactational breast abscesses and underwent percutaneous needle aspiration under local anaesthesia. Data were analyzed by using descriptive statistics.

**Results:**

The estimated incidence of lactational breast abscesses was 0.74% (28/3792). The age range of babies at the onset of breast abscess was 4 to 35 weeks; mean 28.3 ± 10.85 weeks. Forty-four per cent of participants underwent three lactational abscess aspirations and in 24 to 28% of them, it took 8 to 9 days for the abscess to resolve. In 72% of participants, treatment was with needle aspiration plus flucloxacillin. Seventy-six per cent of participants continued breastfeeding after abscess treatment.

**Conclusion:**

The estimated incidence of lactational breast abscess at the Douala General Hospital is 0.74%. Percutaneous needle aspiration under local anaesthesia is an effective treatment for superficial lactational breast abscesses in most cases with or without ultrasound guidance and should be recommended worldwide as first line treatment. Further research is needed to understand the outcome of local infiltration of antibiotics on the abscess cavity.

## Background

Lactational breast abscesses are complications of infectious mastitis and are more frequent among primiparous women. It has been estimated that 0.4 and 3% of women with mastitis develop a breast abscess [1]. A breast abscess is defined as a localized accumulation of infected fluid in breast tissue [2].

Mastitis usually occurs during the first 6 weeks but can occur at any time during lactation [2, 3]. Besides, the incidence of lactational breast abscesses is reducing over time because of the increasing use of antibiotics and improved breastfeeding practices [3]. The most common causative microorganism is *Staphylococcus aureus*, and *methicillin-resistant Staphylococcus aureus* (MRSA) is becoming an increasing problem. Furthermore, other organisms like *Escherichia coli* and *Haemophilus influenza* have been identified [2, 4, 5].

Risk factors for a lactational breast abscess include increasing maternal age at delivery, gestational age greater than 41 weeks, mastitis, primiparity, mother employed outside the home and being married, having breastfeeding difficulties in hospital and having cracked nipples [6, 7]. The diagnosis of lactation breast abscess is clinical and confirmed by an ultrasound scan when available [8, 9].

Antibiotics and incision and drainage have been viewed as standard therapy in managing lactational breast abscesses. Antibiotics of choice such as flucloxacillin 500 mg four times daily orally, or after antibiotic susceptibility testing may be prescribed especially in case of MRSA. Women who are allergic to penicillin may be prescribed Cephalexin but clindamycin is suggested for cases of severe penicillin hypersensitivity. It is noteworthy that MRSA should be presumed to be resistant to treatment with macrolides and quinolones, regardless of susceptibility testing results [2, 10]. More recently, however, there has been an emergence of studies favouring treatment of lactational abscesses with ultrasound-guided needle aspiration, which is considered a less invasive technique that causes less scarring, does not affect breastfeeding, does not require general anesthesia or hospitalization and is less expensive than incision and drainage (I & D). Although I & D has the advantage of breaking down the loculi, if the procedure is carried out under general anesthesia it will also involve hospitalization and regular dressings. This could cause considerable distress to both mother and baby during what can already be a difficult and busy time. Also, I & D is associated with a prolonged healing time, regular dressings, difficulties in breastfeeding, and the possibility of an unsatisfactory cosmetic result and breastfeeding experience [8, 10, 11]. There is a dearth of studies that describe the clinical management of lactational breast abscess in Cameroon.

In Cameroon, the 2011 Demographic Health Survey reported that while 97% of mothers initiate breastfeeding, only 31% of mothers practice exclusive breastfeeding by 0–1 month, 22% by 2–3 monthsand 10% by 4–5 months. The median duration of breastfeeding is 16.5 months [12]. Other Cameroon studies have reported 17.3% [13] and 20% EBF by 6 months and average breastfeeding time of 15.24 months [14].

This study aimed to estimate the incidence of lactational breast abscess and describe its management by percutaneous aspiration at the Douala General Hospital, Cameroon.

## Methods

### Participants and setting

We conducted an observational prospective study from January 1 to October 31, 2015, at the Douala General Hospital (DGH). The DGH is a tertiary care referral institution in Douala with a neonatal intensive care unit and conducts about 1250 births per year. Data was collected from breastfeeding mothers who gave birth at the DGH and returned later because of a breast condition and those referred from other health facilities; Laquintinie Hospital Douala (LHD), a secondary care centre with 2750 births annually and Cite Des Palmier District Hospital Douala (CPHD), a primary care centre with 550 births per year.

### Study population and sampling

We included in the study consenting breastfeeding mothers with normal vaginal delivery of healthy singleton babies at term. The babies had to be normal (normal birth weights and devoid of congenital malformations). Women must have chosen to breastfeed (exclusively or partially). Participants were consecutively enrolled in the study in a non-discriminatory manner as they came to the outpatient department of the Obstetrics and Gynecology Department of the DGH after signing an informed consent form. They were interviewed in English, French or Pidgin English according to the mother’s preference.

Excluded from the study were women with other medical conditions, (diabetes, HIV, renal failure and suspected malignancy), patients with imminent necrosis of skin overlying breast abscess, those who had chosen not to breastfeed or those who did not provide consent.

The following variables were recorded on a pretested interviewer-administered questionnaire: maternal age, gravidity or parity, marital status, educational background, and employment status and smoking history were obtained. Data concerning the place of antenatal care, previous births, whether or not there was nipple damage or pain, history of mastitis/breast abscess in multiparous women, breastfeeding experiences and counselling from healthcare providers. Lactational breast abscesses were diagnosed clinically by the following signs: breast tenderness/pain, redness of any part of the breast, breast lump, and high temperature. Most participants were first diagnosed with breast abscesses more than 3 weeks after giving birth. Milk culture or ultrasonography was not accessible due to cost. However, the clinical findings were disclosed to the participants and we noted the number of breast aspirations, antimicrobial treatment, duration of follow-up, and duration of breastfeeding.

Written informed consent was obtained from the participants and we complied with the Helsinki declaration throughout the study [15].

### Lactational breast abscess aspiration technique

**Fig. 1:**
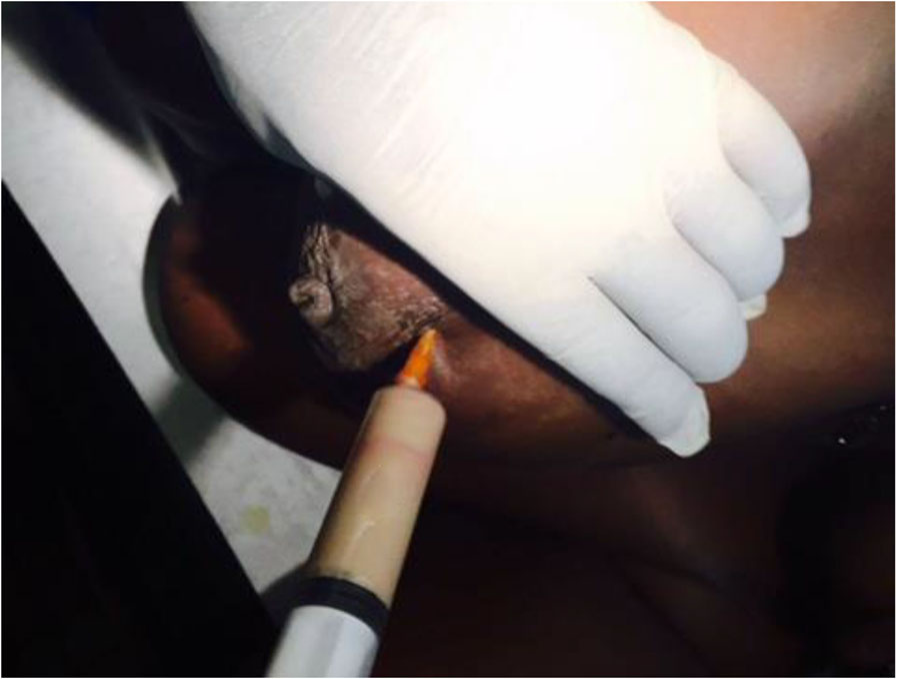
Lactational breast abscess. Aspiration of pus with a 14-gauge needle mounted on a 20 mL syringe

We performed the procedure on an outpatient basis. A 14-gauge needle mounted on a 20 mL syringe was used for aspiration without ultrasonographic guidance. Local anesthesia (lidocaine 2%) was infiltrated at the puncture site using a 29-gauge needle, then a 21 G and finally the 14 G needle. We selected the entry site after cleansing with chlorhexidine to avoid the area of skin thinning if present, where an abscess may drain spontaneously. We aspirated the abscess thoroughly and irrigated the cavity with saline until the aspirate became clear (Fig. 1). This method enabled loculations to be disrupted. We did not send the aspirate to the laboratory for culture because of elevated cost. In some cases, we injected 1 g of ceftriaxone directly into the abscess cavity. Oral flucloxacillin, 500 mg four times a day or erythromycin propionate in case of hypersensitivity of penicillin, was also administered for 10–14 days to these patients. Follow-up of patients was by clinical examination and dressing every 2 days for 1 week and every week for two consecutive weeks, and further aspirations were performed as deemed necessary. We discontinued follow-up when there was no clinical evidence of inflammation and we encouraged patients to continue breastfeeding.

### Data management and statistical analysis

The filled questionnaires were checked for completeness, coded, entered into a Microsoft Excel spreadsheet and exported to the statistical package for the social sciences (SPSS) version 20 for analysis. Data from questionnaires were double entered and merged to check for data entry errors. Categorical variables were summarized as counts and percentages whilst continuous variables were summarized as means and standard deviations.

### Types of intervention

1. Lactational abscess aspiration with oral flucloxacillin
2. Lactational abscess aspiration with oral erythromycin
3. Lactational abscess aspiration with oral flucloxacillin and instillation of ceftriaxone

### Outcome measures

1. Time to resolution of a breast abscess (no recurrence of abscess or need for further intervention). Time was defined in this study as time of presentation for care.
2. Duration of continuation of breastfeeding

This was defined as the totality of duration of breastfeeding both before and after abscess treatment as reported by the participants.

Data were analyzed by using descriptive statistics.

## Results

**Fig. 2:**
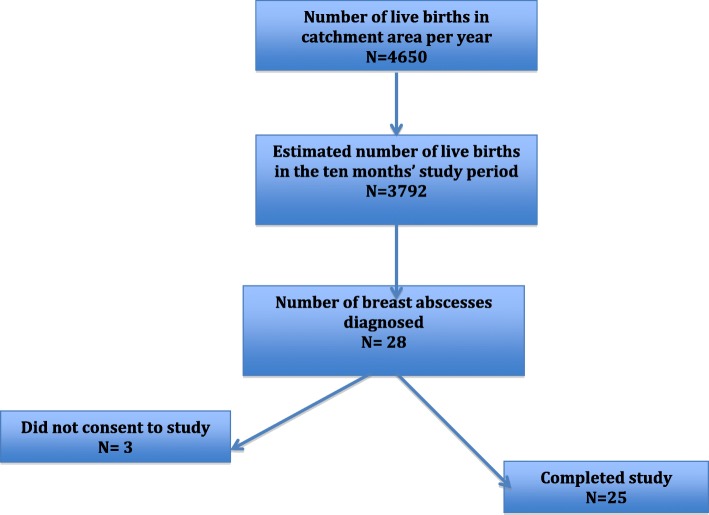
Flow diagram of lactational breast abscess

During the period of study 4550 live births were recorded in the catchment health facilities (DGH, LHD, CPDH) per year. However, the study period was 10 months giving a total of (10/12 of 4550 = 3792) live births. Twenty-eight patients were diagnosed with lactational breast abscess during the study period. Therefore, the estimated incidence of lactational breast abscess in this study period was 0.74% (28/3792). Twenty-five (89.3%) of the 28 patients diagnosed with a lactational breast abscess consented to involvement in and completed the study (Fig. 2).

Table 1 shows that participant’s ages ranged from 17 to 43 years with a mean age of 29.7 ± 7.1 years. The duration of breastfeeding ranged from 12 to 104 weeks with a mean of 45.4 ± 27.8 weeks. The age of babies at the onset of the breast abscess ranged between 4 and 35 weeks with a mean age of 28.33 ± 10.85 weeks.

**Table 1:** Descriptive statistics (*N* = 25)

Characteristics	Frequency or Value
**Maternal Age (Years)**	
Mean	29.72
Standard deviation (range)	7.07 (17-43)
**Baby’s age at the onset of breast abscess (Weeks)**	
Mean	28.33
Standard deviation (range)	10.85 (4-35)
**Duration of breastfeeding (Weeks)**	
Mean	45.36
Standard deviation (range)	27.8 (12-104)

As shown in Tables 2, 56% of participants were less than 30 years old, and most participants were married and multiparous. The highest level of educational attainment for 80% of the participants was secondary school and 12% were unemployed.

**Table 2:** Demographic characteristics of the participants (*N* = 25)

Characteristics	Frequency	Percentage
**Age distribution (years)**		
15–30	14	56
> 30	11	44
**Parity**		
Multiparity	17	68
Primiparity	8	32
**Marital status**		
Married	17	68
Single	8	32
**Level of education**		
Secondary	20	80
Primary	3	12
University	2	8
**Employment status**		
Yes	22	88
No	3	12

Table 3 shows that 44% of participants received three lactational abscess aspirations and it took 8 to 9 days for the abscess to resolve in 24 to 28% of women respectively. Seventy-six per cent of participants continued breastfeeding after abscess treatment. Furthermore, only 16% of participants had prior breastfeeding counselling. Moreover, 29% of multiparous women had a history of mastitis and 72% of participants were treated with oral flucloxacillin and abscess aspiration.

**Table 3:** Clinical characteristics of participants (*N* = 25)

Characteristics	Frequency	Percentage
**Number of aspirations**		
3	11	44
2	8	32
1	6	24
**Duration of follow-up (resolution time)**		
6	4	16
7	4	16
8	6	24
9	7	28
10	4	16
**Continued breastfeeding**		
No	6	24
Yes	19	76
**Breastfeeding counseling received**		
No	21	84
Yes	4	16
**Place of antenatal care**		
Not in the Douala General Hospital	16	56
Others	7	24
Douala General Hospital	4	4
**Past history of mastitis in multiparous women (*N*** **= 17)**		
No	12	71
Yes	5	29
**Type of treatment received**		
Flucloxacillin 500 mg + aspiration	18	72
Flucloxacillin 500 mg + aspiration+ infiltration ceftriaxone	6	24
Erythromycin + aspiration	1	4
**Gestational age at delivery (weeks)**		
38	8	32
39	8	32
40	4	16
41	5	20

## Discussion

This study aimed to estimate the incidence of lactational breast abscess and describe its management by percutaneous aspiration at the Douala General Hospital, Cameroon. The estimated incidence of lactational breast abscess is 0.74% (28/3792). This incidence is consistent with the 0.4% (95% CI 0.14, 0.98) reported by an Australian cohort study [1]. Furthermore, literature reports that 3% of women with mastitis will develop lactational breast abscess [1, 2]. An incidence of lactational breast abscess ranging from 0.19 in 2007 to 0.84% in 2011 [16] has been reported, of which 70.6% were from primiparous mothers with a mean interval from delivery to breast abscess of 41.9 ± 35.8 days. The most frequent risk factors were sore nipples and breast engorgement [16]. The relatively low incidence in this study may be explained by the underreporting of cases of lactational breast abscess and because participants with co-morbidities (diabetes, HIV) were excluded from the study. However, since our diagnosis was mainly clinical, some cases of lactational breast abscesses may have remained undiagnosed. Furthermore, the incidence of lactational breast abscess in the present study is an estimate because the denominator used to calculate it is the number of live births (3792) in the catchment area of the three healthcare facilities during the 10 month study period. This was different from the study by Amir et al. 2004 that followed-up 1193 breastfeeding women by telephone interviews at 6 months whose denominator was exact [1]. The mean age of babies at the onset of breast abscess was 28.3 ± 10.85 weeks. Other studies have reported that mastitis usually occurs during the first 6 weeks but can occur at any time during lactation [2, 10]. Mastitis is a precursor to breast abscess [1, 8]. This is consistent with our study because the high mean infant age of onset of breast abscess may be associated with the weaning period of most of the participants of this study.

### Management of lactational breast abscess

The traditional treatment modality for lactation breast abscesses has been I & D in most settings in both high-income and low-income countries [17]. However, in recent times there is worldwide evidence that aspiration of lactational breast abscesses with or without ultrasound guidance has resulted in good outcomes in terms of an early resumption of breastfeeding [18, 19], cosmetic advantage, less pain and no hospitalization [8, 20, 21].

Eryilmaz et al. 2005 compared a group of 22 patients with lactational breast abscess treated by aspiration without ultrasonographic guidance and another group of 23 patients treated with I & D. They showed that in the I & D group all patients were treated successfully, but one patient (4%) had a recurrence 2 months after complete healing and 16 patients (70%) in this group were not pleased with the cosmetic outcome of I & D. In the needle aspiration group, three patients were treated with a single aspiration and 10 patients (45%) with multiple aspirations, but nine patients (41%) did not heal following needle aspiration and subsequently required I & D too. No recurrences were observed in the needle aspiration group during the follow-up period. In our study six (24%) had one aspiration, eight (32%) had two aspirations, while 11 (46%) had three aspirations. Similarly, O’Hara et al. reported 85% cure rates of 22 abscesses, some of which were aspirated without sonographic guidance especially if abscess was less than 5 cm on clinical examination [22]. Schwarz et al. also reported aspiration without sonographic guidance plus oral antibiotics in 33 patients with a cure rate of 82% [23]. A recent study reported that ultrasound percutaneous guided management of lactational breast abscess was successful for 96% (102/105) of cases regardless of abscess size and allowed continued breastfeeding [20]. We treated difficult abscesses with aspiration and irrigation with saline and infiltration of ceftriaxone 1000 mg in the abscess cavity. The rate of ceftriaxone absorption through the abscess cavity and its bioavailability is not well known. However, bacteria present in the abscess cavity are exposed to high concentrations of the antibiotic injected directly into the abscess cavity. Therefore, use of a large-bore needle, irrigation with saline solution and local infiltration of ceftriaxone appear beneficial in difficult cases because the saline reduces the viscosity of the pus thereby facilitation aspiration and also enables loculations to be disrupted whereas the antibiotic acts directly on microorganisms in high concentrations, although several aspirations were required. Further research is needed to understand the role of antibiotic infiltration in the abscess cavity. Irusen et al. [10] in a systematic review reported a mean time to resolution for women who received a course of antibiotics of 7 days, 7 days for women who received a single dose of antibiotics and 7 days for women who did not receive antibiotics. That notwithstanding, they concluded that there is insufficient evidence to determine whether needle aspiration is a more effective option to I&D for lactational breast abscesses, or whether an antibiotic should be routinely added to women undergoing I&D for lactational breast abscess [10].

### Time to resolution of breast abscess

The resolution time of lactational breast abscesses in this study ranged from 6 to 10 days. However, several studies have reported shorter resolution time of lactational breast abscess among participants receiving treatment with needle aspiration compared with I&D though there was a higher failure rate of treatment in the needle aspiration group [8, 24, 25].

### Continued breastfeeding

In the present study, married women with lactational breast abscess continued breastfeeding for longer periods than single mothers. This is consistent with other studies that reported 87% of participants who continued breastfeeding among those treated with ultrasound-guided aspiration of lactational breast abscess [25]. Primiparous women and single breastfeeding mothers could, therefore, be targeted for extra information in preparation for parenthood classes about breast care thereby avoiding breast abscesses.

### Strengths and limitations of the study

This is the first study in Cameroon, a low-income country that describes the management of lactational breast abscesses by percutaneous aspiration. This may pave the way for other studies that compare the practice of needle aspiration and I & D in Cameroon. This study was hospital-based and only women who consulted the DGH with lactational breast abscess were enrolled for the study. Some abscesses may have been missed because we did not use ultrasonography in the management of the abscesses because it was not cost-effective for most patients. We did not include the sizes of the abscesses, although this would have enabled comparability to other studies. The incidence calculation is an estimate from the number of deliveries in the catchment area during the 10 months of study.

The participants who reported a history of lactational mastitis/abscess in previous births had no supportive medical report to affirm the diagnosis. Finally, we excluded from study women with co-morbidities (caesarean section, preterm birth, HIV-positive and diabetic mothers), which could increase the risk of lactational breast abscess.

## Conclusions

The estimated incidence of lactational breast abscess at the Douala General Hospital is 0.74%. Percutaneous needle aspiration under local anaesthesia is an effective treatment for superficial lactational abscesses in most cases with or without ultrasound guidance. It should, therefore, be recommended worldwide as first line treatment of breast abscess. Further research is needed to understand the role of local infiltration of antibiotics into the abscess cavity.

## Data Availability

“The datasets used and/or analyzed during the current study are available from the corresponding author on reasonable request”.

## Abbreviations

CPDH: Cite des Palmier district hospital
DGH: Douala General Hospital
HIV: Human immunodeficiency virus
I & D: Incision and drainage
IQR: Interquartile range
IWC: Infant welfare clinic
LHD: Laquintinie Hospital Douala
SPSS: Statistical Package for the Social Sciences
